# Ketamine in Status Epilepticus: How Soon Is Now?

**DOI:** 10.3390/neurolint17060083

**Published:** 2025-05-28

**Authors:** Giuseppe Magro

**Affiliations:** Department of Neurology, Lamezia Terme Hospital, 88046 Catanzaro, Italy; giuseppemagro.neuro@gmail.com

**Keywords:** Ketamine, combined polytherapy, Stage 1 Plus, benzodiazepine-refractory status epilepticus, NMDA antagonists, acute etiology in status epilepticus, prolonged status epilepticus, benzodiazepine, status epilepticus

## Abstract

Status epilepticus (SE) is a neurological emergency. Current evidence dictates a step-by-step approach with a first line of therapy consisting of benzodiazepines (BDZs). In many situations, the currently approved approach does not terminate a BDZ-resistant SE. This happens in Stage 1 Plus, a framework designed by the author to recognize cases of probable benzodiazepine-resistant status epilepticus even before treatment initiation. These cases include Prolonged SE (SE lasting > 10 min), the absence of prominent motor phenomena, and acute etiology (primary central nervous system etiologies most of all). BDZ-refractory SE cases (Stage 1 Plus) might require a different approach, one targeting the unresponsive GABA signaling state mediated by NMDA/AMPA receptors, such as combined polytherapy with Ketamine from the start. These considerations stem from the receptor trafficking hypotheses: in prolonged seizure activity and primary central nervous system etiologies, GABA receptors get internalized and move away from synapses, and therefore, SE becomes resistant to BDZ. A rational polytherapy that might restore the unresponsiveness to BDZ in SE should include NMDA antagonists, such as Ketamine. Ketamine has proven effective in many experimental models of status epilepticus, and much evidence is gathering supporting its use in humans, especially in refractory and super-refractory SE. We lack studies evaluating combined polytherapy in SE, especially in the early phases. The author suggests here that Ketamine should be used along with first-line BDZ in the early SE stage falling in the category of Stage 1 Plus and as a first-line anesthetic infusion drug in refractory SE, especially in cases progressing from Stage 1 Plus, eventually adding continuous midazolam/propofol infusion in later phases. This systematic review’s objective is to summarize the presently available evidence of the early use of combined polytherapy that includes Ketamine, along with the currently available evidence of Ketamine use in early, established, and refractory SE. Nine studies were included. Boluses of Ketamine and Midazolam are effective in pediatric convulsive Stage 1 Plus SE. The results show that earlier Ketamine administration (especially within 12 h of SE onset) was significantly associated with improved seizure control, with a more favorable safety profile than Midazolam in refractory SE. Notably, a dosage of less than 0.9 mg/kg/h proves ineffective in terminating SE. Ketamine has the advantage of preventing intubation, possibly shortening the length of stay in the intensive care unit.

## 1. Introduction

Status epilepticus (SE) is a neurological emergency. The incidence of SE reported in the literature ranges from 1.29 to 73.7 cases per 100,000 individuals, varying according to the studied populations and methodological approaches. SE accounts for significant morbidity, mortality, and healthcare resource utilization, with mortality rates ranging from 10% to over 30% depending on etiology, age, and response to treatment. Despite the availability of treatment guidelines, multiple unmet clinical needs persist. These include the lack of reliable tools for the early identification of benzodiazepine-resistant SE, limited therapeutic options that balance efficacy and safety in fragile patients, and the absence of robust evidence supporting optimal timing and combination strategies for second-line therapies. Moreover, the conventional stepwise approach to treatment may delay the initiation of effective interventions in patients with poor prognostic indicators, ultimately impacting outcomes. These challenges underscore the need for a more individualized and mechanism-based approach to SE management that considers the heterogeneity of presentations, etiologies, and pharmacoresistance patterns from the earliest stages [1,2,3]. Many unanswered questions remain regarding SE. For example, we lack approved treatments for the rapid termination of an ongoing seizure that has not progressed to status epilepticus; we do not know if SE represents a failure of unusual seizure mechanism terminations or a different phenomenon from its onset; and we do not know the role of systemic factors and genetics in SE [4].

Currently approved treatment involves a step-by-step sequential approach in which a second drug can only be administered after the failure of the first one [5,6]. The first-phase treatment for convulsive early-status epilepticus is the rapid administration of full doses of benzodiazepines (early SE). The second-phase treatment for convulsive established status epilepticus is a full-loading dose of IV fosphenytoin, levetiracetam, valproic acid, or phenobarbital (established SE) [5]. For the third-phase treatment of convulsive status epilepticus (when a benzodiazepine followed by a single non-anesthetizing antiseizure medication fails to terminate the seizure, or refractory SE (RSE)), clinicians frequently opt for continuous intravenous infusions of anesthetizing antiseizure medications, such as midazolam, pentobarbital, or propofol, rather than administering an additional non-sedating antiseizure drug. This approach is guided by a consensus of experts, which emphasizes the importance of rapidly controlling convulsive status epilepticus to prevent brain damage [7]. However, no Class I randomized controlled trials have been conducted to compare the efficacy and potentially severe adverse effects of continuous infusion anesthetizing medications with those of a second antiseizure medication in the adult population [8]. Super-refractory status epilepticus (SRSE) is defined as SE that continues or recurs 24 h or more after the onset of anesthetic therapy or recurs upon the reduction in or withdrawal of anesthesia [9]. Many studies have explored other options, such as using Ketamine in SE, mostly in refractory [10] and super-refractory SE [11,12]. Ketamine, an N-methyl-D-aspartic acid (NMDA) receptor antagonist discovered in 1962, has become widely used in anesthesia. It provides dose-dependent anesthetic, sedative, and analgesic effects [13]. Its infrequent use in clinical practice is due to its potential side effects, observed in cases of recreational abuse. Concerns about Ketamine use in subjects with epilepsy for possible electrographic seizures following its administration as a sedative agent were not confirmed in later studies [14]. A recent study on Ketamine for refractory SE in neonates and children found it to be effective in about 75% of cases [8]. Additionally, a 2024 systematic review and meta-analysis on the use of Ketamine in pediatric refractory status epilepticus showed SE cessation in 51% of cases with the addition of Ketamine [15]. Ketamine treatment was linked to a reduction in seizures in patients with super-refractory SE. High-dose Ketamine infusions have been associated with lower vasopressor requirements without evidence of an intracranial pressure increase [12]. Clear evidence exists regarding Ketamine’s role in super-refractory SE [12]. Its role in the early SE phase is less consolidated; nonetheless, it is still considered a good choice [5]. Moreover, a recent study on the pediatric population provides evidence for its earlier use as combined polytherapy for early SE [16]. The rationale for the earlier use of Ketamine in SE stems from the receptor trafficking hypothesis. As time passes and seizure activity persists, GABA receptors get internalized and relocate away from synapses; meanwhile, NMDA receptors are upregulated. This is corroborated by observational studies showing how resistance to first-line therapy with benzodiazepine (BDZ) is higher in cases of prolonged seizures [17]. Experimental models show that a similar process happens early in SE, caused by acute processes, especially those primarily involving the central nervous system, thus disrupting brain receptors and network homeostasis [18]. Currently approved first-line therapy involves mainly BDZs, which exert their therapeutic action on GABA receptors. This approach does not always result in seizure control; consequently, a combined polytherapy from the start may be required. In the Established Status Epilepticus Treatment Trial (ESETT), seizure cessation occurred in less than 50% of patients after second-line ASM administration, regardless of whether they received levetiracetam, fosphenytoin, or valproate, following initial benzodiazepine failure [19].

Significantly, delayed benzodiazepine administration (>30–60 min from seizure onset) is associated with a sharp drop in efficacy, with failure rates approaching 70–90% in some cohorts [17]. Etiologies also have a role, with the acute forms showing a lower response to BDZs [20], and disorders of consciousness upon clinical presentation seem to predict the requirement for an anesthetic infusion [21].

Moreover, in children with neurodevelopmental disorders, especially those with co-occurring epilepsy and breathing disorders, the administration of BDZs may worsen respiratory compromise. Therefore, while BDZs are essential in most SE protocols, they are not a solution for all cases. Their use should be moderated by performing individualized risk assessments, particularly in cases of treatment-resistant epilepsy and systemic fragility. The need for tailored approaches, especially early in treatment, highlights the importance of alternative or adjunctive agents, such as Ketamine, for frail patients and those with conditions in which BDZ resistance can be expected.

**Figure 1:**
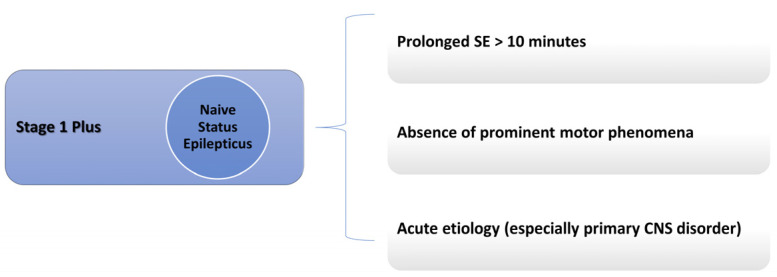
Stage 1 Plus definition.

There are many conditions in which a lack of response to BDZ can be expected: these conditions have been grouped under the umbrella term of Stage 1 Plus in a previous publication by the author [22,23]. These include prolonged SE (a seizure lasting more than 10 min), acute etiologies (mainly primary central nervous system etiologies), and the absence of prominent motor phenomena [22]. However, this last case, such as in non-convulsive SE, might fall in the first category due to the delay in recognition and treatment. SE is considered BDZ-resistant before treatment if it meets at least one of the conditions of Stage 1 Plus. Figure 1 summarizes all Stage 1 Plus treatment-naïve SE cases.

These conditions seem to require a combined polytherapy from the start, one that includes an NMDA antagonist such as Ketamine, and studies are being conducted that favor this hypothesis. Moreover, Ketamine might help prevent systemic complications, such as supporting blood pressure [4]. This work builds on the original proposal of Stage 1 Plus, a framework designed by the author to categorize cases of probable benzodiazepine-resistant status epilepticus even before treatment initiation, and highlights the currently available evidence for early treatment with the NMDA antagonist Ketamine. The author suggests here that Ketamine should be used along with a first-line BDZ in the early SE stage falling in the category of Stage 1 Plus (immediately in every case of SE with acute primary central nervous system etiologies and in cases of SE lasting more than 10 min) and as a first-line anesthetic infusion drug, especially in those cases of Stage 1 Plus progressing to refractory SE, while eventually adding continuous midazolam/propofol infusion in later phases (potentially restoring patient response to a BDZ-resistant SE). This systematic review aims to provide pathophysiological evidence and summarize the evidence for the early use of Ketamine in SE, focusing on the currently available evidence for early combined polytherapy with Ketamine and the most robust evidence existing in the literature regarding Ketamine in refractory SE (RSE).

## 2. Methods and Research Outputs

**Figure 2:**
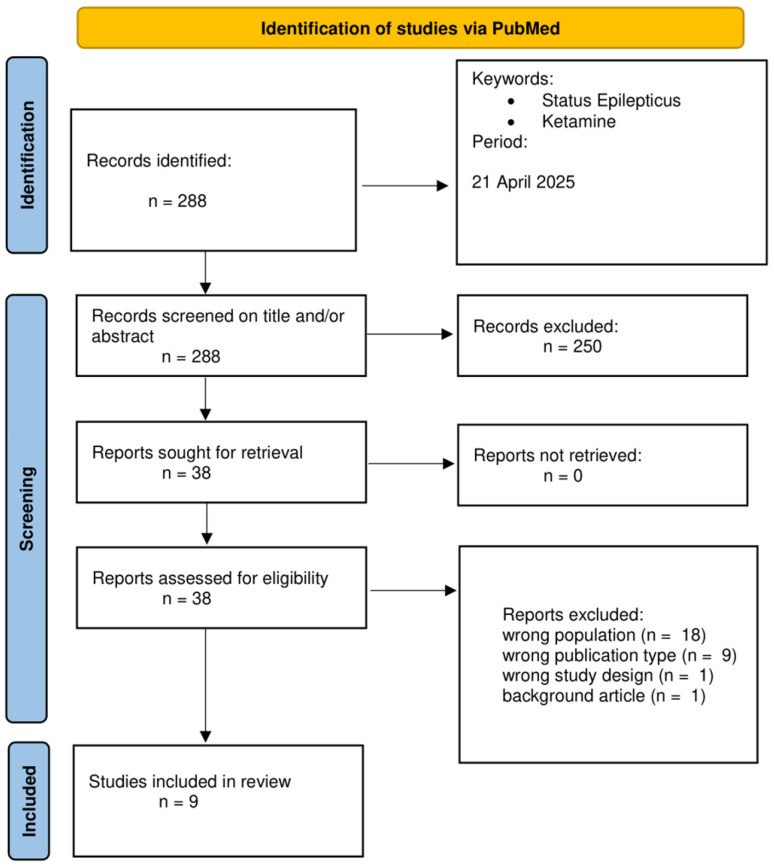
PRISMA flowchart.

PubMed was searched with no restriction on time, including publications up to 21 April 2025. The search terms and the string used were as follows: “ketamine” AND “status epilepticus”. The research yielded 288 results. English language restriction was applied. Case reports and series with fewer than 25 patients, studies from pre-hospital settings, and super-refractory SE studies were excluded. This is due to many confounding factors and high variability of studies in out-of-hospital settings, which have substantial heterogeneity in protocols, delayed treatment documentation, and uncontrolled environments that limit interpretability. Studies on SRSE were also excluded, since Ketamine is already widely used and accepted in this context and its role is less debated. Moreover, in super-refractory cases in which Ketamine is used, many confounding factors intervene, such as numerous drugs being given before and during Ketamine infusion, making it harder to extrapolate Ketamine’s role. Consequently, only articles of Ketamine in early, established, and refractory SE were included in the analysis. Reviews and studies on animals were excluded from the primary analysis, and the output table summarizes all studies. The following variables were collected for each study: population, SE type, Ketamine timing, initial dose, infusion rate, response rate, adverse events, population group, median age, pre-existing epilepsy, and anti-seizure medication before Ketamine. Studies in which super-refractory SE and RSE patients were not differentiated were excluded and classified as having “wrong study design”. Pre-hospital works and small case series were excluded and classified as having “wrong population”. Reviews and meta-analyses were excluded and classified as being “wrong publication type”. A total of 9 articles are included in Table 1, which outlines all the most important and robust evidence from human studies about Ketamine in SE in earlier phases, such as early, established, and refractory SE. In Figure 2, the PRISMA flowchart summarizes the screening process.

### Risk of Bias Assessment

The ROBINS-I tool (Risk Of Bias In Non-randomized Studies of Interventions) was used to evaluate the methodological quality of the eight non-randomized studies included. One randomized controlled trial (study number 1 in Table 1) was assessed separately using the RoB 2.0 tool and was deemed to have a low risk of bias. Among the non-randomized studies, five were rated as having a moderate risk of bias, primarily due to potential confounding variables, lack of randomization, and non-standardized intervention protocols (numbers 2, 3, 4, 5, and 9). Two studies (numbers 6 and 8) were assessed as having a serious risk of bias due to retrospective design, variability in intervention timing/dose, and absence of comparator groups. After considering its prospective design and systematic outcome documentation, one study (number 7) was classified as having moderate risk. The most common concerns included confounding variables due to SE etiology or severity, selection bias, and outcome reporting bias. See Table 1 for references to the studies’ numbers.

## 3. Evidence for Early Use of NMDA Receptor Antagonists

### 3.1. Trafficking of GABA Receptors and Etiology

**Figure 3:**
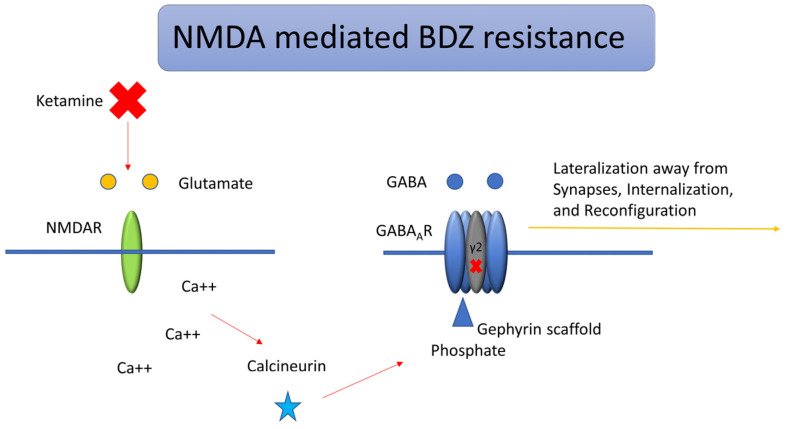
Lateralization and BDZ resistance of GABA-A receptors mediated by NMDA. During status epilepticus, activation of NMDA receptors modulates downstream kinase and phosphatase pathways that govern the trafficking of GABA-A, AMPA, and NMDA receptors. Calcium influx through NMDA or calcium-permeable AMPA receptors activates the phosphatase calcineurin, leading to the dephosphorylation of the gamma (γ2) subunit of postsynaptic GABA-A receptors. This modification reduces their affinity for the inhibitory scaffolding protein gephyrin, resulting in receptor dispersion from synapses toward endocytic sites, where the AP2 adaptor complex facilitates internalization via clathrin-mediated endocytosis. Ketamine exerts its action by blocking NMDA receptors and potentially reversing BDZ resistance.

The rationale for Ketamine in SE stems from the potential role of NMDA receptor antagonists, which could potentially revert a BDZ refractory state. This happens because GABA-A resistance seems to be mediated and amplified by the overexpression of NMDA receptors [17,24,25]. Many mechanisms are involved in SE resistance to first-line therapy with BDZ. Among these, time itself is the most significant predictor of treatment resistance, following the dynamic changes in GABA-A receptor trafficking that occur during prolonged seizure activity. Benzodiazepines favor chloride influx by binding to Cl-permeable GABA-A receptors and increasing channel conductance. BDZ has a hyperpolarizing effect because the intracellular space has a lower chloride concentration. Many changes take place during prolonged seizure activity. The following are the most significant ones: GABA-A receptor Cl- gradient inversion (due to the failure of active Cl-transport out of the cells, mostly mediated by NKCC2), GABA-A receptor internalization, GABA-A receptor trafficking away from synapses, and the loss of high-BDZ-affinity gamma-subunits [17,22,25,26]. Many experimental models have shown how GABA-A receptor trafficking occurs very early in SE (10 min). The gamma2 subunit of GABA receptors is found mainly at synapses, and those are the ones with a higher affinity to BDZ [27,28]. The activity-dependent entry of Ca++ through NMDA and AMPA receptors leads to a dephosphorylation of the gamma2 subunit, consequently leading to trafficking of the high-BDZ-affinity subunit away from synapses and the loss of BDZ response in SE [29]. As time passes, chloride increases its concentration inside the cell, which causes a shift of GABAergic signaling from inhibitory to excitatory: ongoing seizure activity favors the expression of NKCC1, which favors Chloride influx [30], whilst the surface expression of KCC2, which favors Chloride efflux, is reduced [31,32]. As a consequence, GABA signaling will become excitatory. These dynamic changes, with increased internalization and lateralization of GABA receptors away from synapses, were observed as early as 10 min from SE onset [33,34]. The term lateralization, in the context of GABA-A receptor trafficking, refers to the activity-dependent relocation of synaptic receptors away from the postsynaptic density toward extra-synaptic membrane domains, where they become functionally less effective. This process is triggered by sustained NMDA and AMPA receptor activation during prolonged seizure activity, leading to calcium influx and the activation of intracellular phosphatases such as calcineurin and protein phosphatase 2B [34,35]. These enzymes dephosphorylate critical residues on the gamma2 subunit of GABA-A receptors, thereby disrupting their anchorage to the gephyrin scaffold at inhibitory synapses. Once detached, receptors are not immediately internalized but move laterally along the membrane, drifting away from the synaptic cleft where the GABA release is concentrated. This lateralization causes a functional decoupling between GABAergic neurotransmission and receptor activation, significantly reducing the efficacy of benzodiazepines, which require synaptic gamma2-containing GABA-A receptors for optimal binding and modulation. The process precedes and accompanies receptor internalization and is a key contributor to the early loss of inhibitory tone and the emergence of pharmacoresistance (BDZ resistance) in status epilepticus [35,36]. Figure 3 summarizes the lateralization of GABA-A receptors mediated by NMDA and Ketamine’s role.

Moreover, GABA trafficking is mediated by NMDA and AMPA overexpression. While BDZ loses its efficacy late in models of SE, AMPA and NMDA receptor antagonists retain their efficacy even in late models of SE and might help restore the unbalanced excitatory versus inhibitory signaling [26]. Neuroinflammation and increased levels of drug-metabolizing enzymes at the blood–brain barrier also play a role in resistance to anti-seizure medications (ASMs) during SE [34]. Resistance to BDZ increases with time, but not every SE develops resistance to BDZ. Therefore, other factors must be at play. Among these, model-dependent differences in SE support the hypotheses that etiology must play an important role [18].

Both time and etiology are factors at play, according to substantial evidence from human studies. A study comparing low- and high-income nations concerning treatment latency made it evident that when latency to treatment in SE reaches 60 min, resistance to first-line BDZ therapy in convulsive SE can be as high as 89% [17]. The most critical etiologies associated with a worse outcome and a lower chance of seizure termination are acute etiologies, mainly primary central nervous system (CNS) disorders [20]. Also, non-convulsive semeiology seems to bear a negative outcome; whether this is due to a delay in recognition remains to be elucidated [20]. Acute primary CNS, acute secondary CNS, progressive SE, age, and impaired consciousness were all substantially linked to a higher odds of death, according to a logistic regression model that controlled for age, SE semeiology, and consciousness before treatment initiation [37].

That is why the author previously proposed a definition of Stage 1 Plus to identify cases of naïve SE that might be unresponsive to BDZ and, therefore, require a different approach. Stage 1 Plus is a treatment-naïve SE stage associated with BDZ resistance, with prolonged seizure activity (≥10 min), acute etiologies, and prevalent non-motor phenomena [22,23]. These cases benefit from earlier NMDA receptor antagonists (such as Ketamine), possibly requiring a combined polytherapy approach. A combined approach, one that includes BDZ and NMDA receptors antagonists, cannot be abandoned for the time being for two reasons: we currently lack clinical trials evaluating early therapy in SE with NMDA receptors antagonists only, and NMDA receptors antagonism might help restore SE response to BDZ.

When it comes to resistance to ASMs, it must be considered that some individuals carrying single-nucleotide polymorphisms (SNPs), for example, SNPs in adenosine triphosphate (ATP)-binding cassette (ABC) genes, can also increase the risk of treatment resistance to ASMs. Evidence has shown that this gene can change treatment responses and make the blood–brain barrier less permeable to psychotropic medication [38].

### 3.2. Model of SE Response to NMDA Antagonists

Numerous experimental models of SE have demonstrated the effectiveness of Ketamine in seizure control. The time-related decrease in conventional antiepileptic drugs’ effectiveness during RSE may result from early maladaptive synaptic GABA-A receptor internalization and NMDA receptor externalization. Addressing these receptor changes can stop SE, as shown in the famous model of dual therapy by Niquet et al. The combination of midazolam and Ketamine was more effective in reducing multiple indicators of SE severity compared to either a double dose of midazolam or Ketamine alone and to combinations of valproate with midazolam or Ketamine. These findings suggest a synergistic interaction between midazolam and Ketamine. Furthermore, this dual therapy also lessened acute neuronal damage and the later development of epilepsy following SE [39]. This was also shown in the triple therapy experiment by the same authors, in which the midazolam–Ketamine–valproate combination was more efficient than triple-dose midazolam, Ketamine, or valproate monotherapy or higher-dose dual therapy in reducing several parameters of SE severity in prolonged SE [40]. Antiseizure and neuroprotective properties in chemically induced seizures have been extensively described, along with functional improvement, neuroinflammation reduction, and cognition preservation [41].

A recent study showed how the combination of low-dose Ketamine and propofol achieved rapid seizure control and was as effective as a high dose of propofol alone. The study concluded that effective seizure suppression can be obtained using a low-dose Ketamine–propofol combination in an experimental model of RSE, potentially avoiding the adverse effects of high-dose monotherapy [42]. The impact of time has been extensively studied in a randomized controlled trial with a rat model, which showed how diazepam–Ketamine dual therapy at 10 min, 20 min, and 30 min from the beginning of SE terminated seizures and achieved high survival rates [43]. Regarding toxicity, NMDA antagonists have been implicated in toxic changes in rat brains, such as neuronal vacuolization [44]. As of now, these changes have not yet been described in humans. There are additional concerns regarding the developing brain, for which (in rats and primates in the neonatal period) NMDA receptor antagonists may have long-term consequences [45,46].

### 3.3. Other NMDA Receptor Antagonists

Currently available NMDA receptor antagonists, in addition to Ketamine, include magnesium sulfate, dizocilpine, amantadine, and dextromethorphan [47]. Nonetheless, their applicability in clinical practice and safety profile remains to be seen. Ketamine remains the only NMDA antagonist with substantial evidence of clinical efficacy in human status epilepticus, particularly in refractory and super-refractory cases.

Magnesium sulfate has been used in the context of eclamptic seizures. In a major randomized controlled trial, it was proven effective in controlling SE due to eclampsia [48]. Its effects are primarily attributed to the blockade of NMDA receptor ion channels, thereby preventing the influx of Ca^2+^. However, the evidence supporting intravenous magnesium sulfate administration for non-eclamptic SE or RSE is classified as Oxford Level 4, Grade D. Therefore, its routine use in these patients is not recommended until further prospective studies confirm its effectiveness [49].

Amantadine is a low-affinity, non-competitive antagonist of NMDA receptors. Its primary mechanism involves enhancing dopamine release and reducing dopamine reuptake within the central nervous system. It was demonstrated that amantadine exerts its NMDA receptor-blocking effect by accelerating channel closure [50].

Other NMDA receptor antagonists that have demonstrated variable antiseizure activity, mainly in experimental models, include Memantine [51], Dextromethorphan [52], and Dizocilpine [53]. Furthermore, compounds like ifenprodil, felbamate, remacemide, and riluzole have demonstrated partial NMDA antagonism and some efficacy in experimental seizure models, yet none have been evaluated in clinical SE settings [47]. In contrast, Ketamine is the only NMDA antagonist consistently studied in SE across multiple human cohorts, including randomized trials in pediatric populations. It can be administered as both a bolus and continuous infusion, and it is already widely used in anesthesia and critical care for its cardiovascular stability and safety in respiratory function. It also offers potential neuroprotective effects by reducing excitotoxicity, which may be especially relevant in prolonged or severe SE. For all these reasons, Ketamine currently remains the most viable and clinically relevant NMDA antagonist for use in SE. While future studies may broaden the therapeutic landscape, Ketamine is, at present, the best-positioned agent in this class for emergency seizure management.

### 3.4. Translational Challenges from Animal Models to Clinical Application

While preclinical studies have been instrumental in elucidating the mechanisms of pharmacoresistant SE and demonstrating Ketamine-based polytherapy’s efficacy, significant translational limitations must be acknowledged. First, species differences in receptor distribution, subunit composition, and neuronal network organization can markedly influence drug response, limiting the generalizability of results. For example, the density and trafficking dynamics of GABA-A and NMDA receptors in rodents differ from those in humans, particularly during developmental stages [35,54]. Second, dosing discrepancies are notable: animal models often rely on higher or repeated doses of Ketamine to induce therapeutic effects, which may not be directly translatable to safe clinical regimens in humans [39]. Third, the heterogeneity of SE models—ranging from pilocarpine-induced seizures to kainic acid or cholinergic paradigms—reflects varied pathophysiologies that may not accurately mirror human SE, particularly concerning etiology, comorbidities, and seizure semiology [55]. Moreover, most animal studies evaluate treatment initiation within a controlled time frame, while in clinical practice, delays in diagnosis and treatment are common and significantly impact treatment response. These differences underscore the need for a cautious interpretation of preclinical data and highlight the urgency of well-designed human trials to validate early Ketamine use in SE.

## 4. Ketamine: Properties and Advantages

### 4.1. Properties

Ketamine is a non-competitive NMDA receptor antagonist. Two enantiomers are recognized: the S (+) dextrogyre Ketamine, a potent analgesic drug, and the R (−) levogyre Ketamine, which is linked to the unpleasant reaction observed during the emergence phase [41]. Ketamine blocks the NMDA receptor through a mechanism known as open-channel trapping; it has high fat solubility and a low plasma protein binding rate, which results in rapid entry into the blood–brain barrier with a fast onset of action. The maximum plasma concentration time ranges from 1 to 5 min when given intravenously. Its half-life is 3.6 h. Loss of consciousness occurs within 45 s and lasts 10–15 min, following a single dose, typically 1–2 mg/kg over 1 min. Analgesia lasts for 40 min and amnesia for 1–2 h. Electroencephalographic (EEG) changes induced by Ketamine comprise alternating slow-delta and gamma oscillations [56]. Following Ketamine administration, heart rate and systolic blood pressure increase [14]. The advantages of Ketamine as the ideal anesthetic include cardiovascular and respiratory stability, especially in cases of trauma, burns, and shock [41]. Its safety is well established in children, for whom it is considered a safe choice during painful procedures; however, its use in status epilepticus—especially in patients with pre-existing cardiorespiratory compromise or QT prolongation—requires careful consideration and individualized risk/benefit assessment [57]. Concerns have been raised regarding a possible increase in intracranial pressure, which does not appear to be clinically relevant. Ketamine is now safely used in traumatic brain injuries and nontraumatic neurological diseases [12,58,59]. On the other hand, heart rate and arterial blood pressure can rise due to catecholamine reuptake blockage and increased sympathetic activity. Supraventricular tachycardia and atrial fibrillation have been rarely reported [60]. A clear advantage of Ketamine is its safety from a respiratory standpoint: respiratory depression is rare, skeletal muscle tone is maintained during anesthesia, and pharyngeal and laryngeal reflexes are retained [14,61]. Many authors believe the most advantageous thing about Ketamine is that it prevents hypoxemia and endotracheal intubation in SE [62].

Ketamine’s therapeutic effects in status epilepticus may, in part, involve brain-derived neurotrophic factor (BDNF) signaling via TrkB activation—a pathway known to mediate Ketamine’s antidepressant effects and exert neuroprotective roles in epilepsy through the modulation of synaptic plasticity and neuronal survival [63].

### 4.2. Adverse Effects Associated with Ketamine and Frail Patients

A recent systematic review highlighted how the occurrence of more severe adverse effects may be linked to the patients’ underlying conditions or overall poorer health status, rather than being directly caused by Ketamine. Milder side effects were transient and self-limiting, resolving after treatment without recurrence [64]. Nonetheless, caution should be taken in vulnerable patients.

Several publications have reported adverse effects associated with Ketamine use, including sialorrhea, hepatotoxicity, cholestasis, cardiac arrhythmias, and metabolic acidosis. In patients with unstable cardiovascular disease, QT prolongation, heart failure, advanced hepatic impairment, or severe renal dysfunction, Ketamine should be administered with great caution, especially if there is a risk of drug accumulation or adverse pharmacodynamic interactions. Likewise, in patients with acute delirium, psychosis, or a history of dissociative disorders, Ketamine’s neuropsychiatric effects may exacerbate underlying symptoms [60,65,66]. Therefore, caution should be adopted towards frail patients with a history of hepatic disease, cardiac arrhythmias, and kidney disease. Ethical concerns regarding Ketamine’s early use arise in these situations. Nonetheless, being a second-line anesthetic agent, attributing adverse effects to Ketamine alone proves challenging.

Ketamine’s long-term consequences are mainly known from abuse settings; whether this applies to patients receiving Ketamine in SE and how much this is solely attributable to Ketamine alone in SE remains to be seen. Chronic recreational Ketamine use has been linked to reduced gray matter volume, decreased white matter integrity, and diminished functional connectivity between thalamocortical and corticocortical regions. These structural and functional brain alterations may underlie some of the long-term cognitive and psychiatric effects observed in Ketamine users, including memory deficits and impaired executive function [67]. More studies are needed in the earlier phases of SE to understand the role of Ketamine. Starting Ketamine for neuroprotection earlier than its current use as an anesthetic drug late in refractory status epilepticus could mean preventing long-term consequences that are strictly related to glutamatergic damage [68].

## 5. Discussion of Available Evidence

Table 1 summarizes the studies included in the review.

**Table 1:** This table summarizes the most robust evidence of early use of Ketamine in status epilepticus.

No.	Author	Population	SE Type	Ketamine Timing	Initial Dose	Infusion Rate	Response Rate	Adverse Events	Median Age	Pre-Existing Epilepsy	ASMs Before Ketamine
1	Othman et al., 2025 [16]	Children (144)	Early SE	Immediately (34 min)	2 mg/kg bolus	NA (Bolus only)	76% (Keta-Mid) vs. 21% (Pla-Mid)	12.5% (Ket-Mid) vs. 35.7% (Pla-Mid)	2.5 years	24%	None
2	Jacobwitz et al., 2024 [69]	Pediatrics (117)	RSE	11.8 h	1 mg/kg	1–6 mg/kg/h	61% (Keta) vs. 28% (Mid)	3% (Keta) vs. 24% (Mid)	0.8 years	28%	None
3	Horvat et al., 2025 [10]	Pediatric cardiac patients (34)	RSE	24.7 h	NA	10 mg/kg/h	73% Keta vs. 63% Mid	54% Keta 68% Mid (No difference)	0.24 years	32%	2+
4	Jacobwitz et al., 2022 [70]	Neonates & children (67)	RSE	20 h	1 mg/kg	0.5–7 mg/kg/h	46% cessation (28% reduction)	4%	0.7 days	25%	3
5	Fletman et al., 2024 [71]	Adults (73)	RSE	Concurrent with midazolam (27 h)	1.5 mg/kg	1.2–10 mg/kg/h	Improved SE duration	None noted	57 years	36%	4
6	Harnicher et al., 2024 [72]	Adults (51)	RSE	44.8 h	NA	0.6–3 mg/kg/h	43% full cessation	5.9%	58 years	13%	4
7	Gaspard et al., 2013 [60]	Mostly Adults (60 episodes, 46 adults, 12 children)	RSE	<12 h likely response to Ketamine, >10 days no response	1.5–5 mg/kg	2.75–10 mg/kg/h	57% total; 32% Ketane-attributed	7%	24 ears	15%	6
8	Kimmons et al., 2024 [73]	Adults (28)	RSE	19.6 h response, 36 h no response	1 mg/kg	0.6–2 mg/kg/h	71.4%	Hpertension 39.3%, Hypotentsion 31.8%	62 years	53.6%	3
9	Srinivas et al., 2023 [74]	Mostly Adults (9 adults, 2 children)	RSE	2.8 days	2–10 mg/kg	2.43–6.66 mg/kg/h	60.5%; 44.4% attributed to Keta	28.4% Hypertension; 11% Hypotension	55 years	34.6%	3

ASM: anti-seizure medication. RSE: refractory status epilepticus. NA: not available. Keta: Ketamine. Mid: Midazolam.

Most evidence comes from retrospective studies; only one study by Othman et al. is a randomized controlled trial of Ketamine + Midazolam as a first-line bolus therapy versus Midazolam in the pediatric population, with prompt administration in the early SE less than 30 min from seizure onset [16]. All the other studies are retrospective studies and case series. All of these studies involve cases of refractory SE and the Ketamine continuous infusion protocol. Only one study by Fletman et al. was designed to evaluate the combined use of Midazolam and Ketamine with continuous infusion. In most refractory SE studies, a bolus is usually administered, followed by continuous infusion [71]. The bolus dosage from all the studies ranges from 1 to 10 mg/kg; most studies adopt a 1–2 mg/kg dosage for the initial bolus. The rate of continuous Ketamine infusion ranges from 1 to 10 mg/kg/h, with the value of 2–3 mg/kg/h used in most cases. Notably, a dosage of less than 0.9 mg/kg/h proves ineffective in terminating SE, as shown in the study by Gaspard et al. [60]. All these studies on RSE prove that earlier Ketamine administration (especially within 12 h of SE onset) is significantly associated with improved seizure control [70,73]. Most of the studies show benefits for administration within 24 h. On the other hand, late administration proves to be ineffective: Ketamine initiated after 10 days of SE onset showed minimal to no benefit [60]. Regarding etiologies, having a structural epilepsy or an inflammatory/infectious underlying cause of SE is associated with a better response to Ketamine [60,73,74], probably due to a greater contribution of glutamate-mediated excitotoxicity. Regarding anoxic etiologies, while some studies found no statistical difference in response to Ketamine of anoxic versus non anoxic etiology [71], having an anoxic brain injury as the underlying cause was associated with a significantly lower overall rate of SE cessation following treatment with Ketamine and other agents, with a 91% reduction in the likelihood of achieving cessation in the study by Srinivas et al. [74]. In the same study, a significant predictor of negative response to Ketamine was SE duration. Different studies show different responses to Ketamine in the anoxic state [72]. The response of anoxic status epilepticus to Ketamine appears to be heterogeneous: while earlier multicenter studies reported minimal efficacy, more recent cohorts suggest that early, high-dose Ketamine administration may achieve seizure control in a subset of post-anoxic patients, particularly when the extent of cortical injury is limited. A comparison analysis of Ketamine versus Midazolam favors Ketamine as a first-line anesthetic infusion in pediatric RSE [69]. Concerns have been raised over the years regarding Ketamine use in trauma due to a possible increase in intracranial pressure (ICP). An intracranial pressure monitor was used in a study to monitor patients with existing intracranial hypertension, whose ICP remained unchanged after Ketamine initiation [74]. As for adverse events, these seem to be related more often to Midazolam than Ketamine [74]. Adverse events are mainly hypertension and hypotension following Ketamine administration [60,69,71,73,74]; nevertheless, the safety of Ketamine has been established in children with cardiac disease too [10]. Adverse effects are more thoroughly discussed in a later section. Only one study evaluated the continuous infusion of both Midazolam and Ketamine, thus proving the potential benefits of combined polytherapy from the start: the odds of RSE lasting more than 24 h and therefore becoming super-refractory SE were significantly lower in the combined (ket + MDZ) group versus the monotherapy group. Moreover, patients with a greater disease severity favored the combined polytherapy [71]. This aligns with the hypotheses that primary CNS disorders might need combined polytherapy from the start, falling in the category of Stage 1 Plus [22,23]. Moreover, Ketamine seems to prevent intubation, which is an advantage in preventing related infections and reducing the length of stay in intensive care units [73]. The highest quality of evidence comes from the trial recently published by Othamn et al., which evaluated combined polytherapy as the first line of treatment in SE in generalized convulsive SE in children, who were equally randomized to receive a Ketamine bolus plus midazolam (Ket + Mid) or a placebo plus midazolam (Pla + Mid) [16]. In this study, the cessation of clinical seizures at five minutes occurred in 76% of children in the Ket + Mid group compared with 21% in the Pla + Mid group. Children with primary CNS disorders, such as mass lesions and traumatic brain injury, were excluded. In the study, children mostly fall in the category of what has been defined as Stage 1 Plus, mostly for prolonged seizure activity rather than a primary CNS disorder. No benefit was found for combined therapy based on different etiologies. In a study by Lattanzi et al., the primary CNS etiology was associated with a worse outcome [37], whilst in the study by Llauradó et al., status epilepticus secondary to an acute brain injury responded poorly to BDZ [20]. As already discussed, this is also shown in mouse models of acute brain injury SE. Therefore, it is possible they did not find any statistical difference in etiologies because of the study’s design. The work reports a high percentage of children with seizures lasting > 30 min; this could represent a possible selection bias. Such a high percentage of patients reaching the ER this late without being previously treated is not as frequent in the US and Europe. This might mean that the population of the study was intrinsically more susceptible to respond to combined polytherapy; nonetheless, this does not diminish the crucial results of the study, which might represent a true milestone in status epilepticus treatment in children, possibly changing the outcome and disability burden of many children. We currently lack clinical trials evaluating an earlier combined approach, including Ketamine, in the adult population. The only study that currently evaluates combined polytherapy in the adult population is the one by Fletman et al., which provides evidence in favor of combined Ketamine and Midazolam infusion polytherapy in RSE [71]. Another interesting aspect of Ketamine is its ability to reduce interictal continuum activity, which is the EEG correlate of a significant predisposition to develop ongoing seizures [72]. A previous systematic review of data on 244 adults with refractory SE receiving at least one continuous infusion anesthetic revealed a 74% termination rate after starting Ketamine [75]. More studies evaluating an earlier combined approach are needed.

### 5.1. Adverse Effects Reported in the Included Studies

In the nine studies reviewed, reports of adverse events were inconsistent but typically rare. In Othman et al.’s randomized controlled trial, the incidence of adverse effects, such as the requirement for endotracheal intubation, was significantly lower in the Ketamine-plus-midazolam group compared to the midazolam-only group (4.2% vs. 20.8%) [16]. In the large pediatric retrospective cohort by Jacobwitz et al., Ketamine was associated with fewer adverse events than midazolam when used as a first-line anesthetic, with a reported incidence of 3% versus 24%, respectively [69]. Similarly, in a separate single-center cohort of neonates and children, only 4% of patients experienced short-term adverse events attributable to Ketamine, including hypertension and delirium [70]. In the adult population, Kimmons et al. reported hypotension in 31.8% and hypertension in 39.3% of non-intubated patients receiving Ketamine. However, these events were generally tolerated and did not correlate with infusion duration or dosing [73]. Gaspard et al. found that Ketamine was discontinued due to possible adverse events in 5 of 60 episodes (8.3%), though these were primarily attributed to concurrent anesthetic agents rather than Ketamine itself [60]. In the cohort analyzed by Srinivas et al., fluid overload was observed in 28.4% of patients, but no significant cerebral hemodynamic complications were reported [74]. Notably, in Horvat et al.’s pediatric cardiac population, the incidences of adverse events attributable to Ketamine or midazolam were equivalent, suggesting a comparable safety profile in this hemodynamically vulnerable cohort [10]. Collectively, these findings suggest that Ketamine, when used for the treatment of refractory status epilepticus, is generally well tolerated with a lower or comparable rate of adverse events compared to midazolam, especially in pediatric populations. However, specific hemodynamic effects such as transient hypertension or fluid overload require careful monitoring, particularly in adult and non-intubated patients.

### 5.2. Limitation of Generalizability

Most of the available evidence of early Ketamine administration comes from pediatric studies, which raises considerations regarding their generalizability. Pediatric and adult SE differ in etiology, pathophysiology, pharmacokinetics, and pharmacodynamics. For instance, children generally exhibit more robust NMDA receptor expression, altered drug metabolism, and divergent cortical excitability patterns compared to adults. This is especially true in neonates, who have a completely different receptor framework [76]. Moreover, the safety profile and hemodynamic tolerance of Ketamine may vary with age and comorbidities, which are more prevalent in adult patients.

### 5.3. How Soon Can It Be Administered?

**Figure 4:**
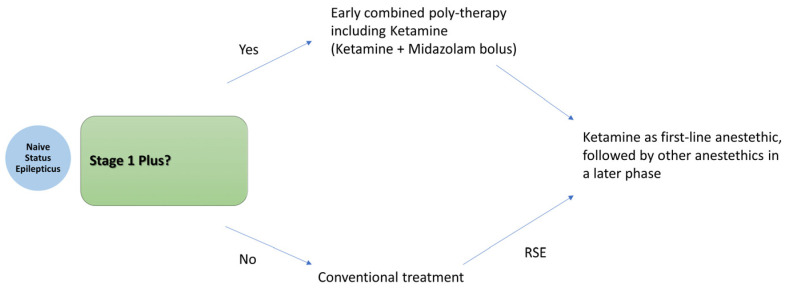
Proposed treatment algorithm for early Ketamine plus Midazolam boluses in Stage 1 Plus.

The existing literature supports the early administration of Ketamine in status epilepticus. The optimal timing, however, depends on the clinical context and is guided by the proposed treatment algorithm. We recommend an early combined polytherapy approach—administering a Ketamine bolus alongside midazolam—in cases of likely benzodiazepine-resistant treatment-naïve SE, defined here as Stage 1 Plus. This includes patients presenting with prolonged seizures lasting more than 10 min, a non-convulsive semiology, or a primary central nervous system etiology. This strategy mirrors the approach adopted in the pediatric trial by Othman et al. In all other cases, particularly in Stage 1 Plus progressing to refractory SE, early Ketamine administration as the initial anesthetic agent of choice is suggested. The overall treatment strategy is illustrated in Figure 4.

## 6. Conclusions and Future Directions

These works of combined polytherapy bring up essential considerations: lower doses might be required to control SE with fewer adverse events. Moreover, Ketamine has the enormous advantage of preventing intubation, possibly shortening the length of stay in the intensive care unit and preventing intubation-related infections. Some conditions, such as prolonged SE (>10 min) and primary central nervous system etiologies (Stage 1 Plus), seem to require a different approach, such as combined polytherapy from the start, especially one counteracting the unbalanced networks of NMDA/GABA receptors, such as with Ketamine. This drug’s mechanism of action offers potential in restoring responsiveness to GABAergic therapies in SE. Future studies should help validate its earlier use in the adult population, which we currently lack in the early stages, and in cases of established SE. Combined polytherapy from the start, in those situations in which a loss of GABA signaling can be expected (Stage 1 Plus), might represent a better choice and possibly change the outcome and disease burden for many people. Ketamine polytherapy with first-line BDZ in the early SE stages falling in the category of Stage 1 Plus and as a first-line anesthetic infusion drug in those cases of Stage 1 Plus progressing to refractory SE might represent a more reasonable approach in cases that are probably BDZ-resistant. The current lack of high-quality adult trials investigating Ketamine for SE represents a significant gap in the literature. To address this, we propose that future research prioritize the development of multicenter, prospective randomized controlled trials designed explicitly for early or established-phase SE, rather than focusing solely on super-refractory cases. These studies should incorporate the following: dose-finding designs that compare both bolus and continuous infusion strategies; time-to-treatment stratification to evaluate whether early administration improves outcomes, as seen in many retrospective studies; standardized endpoints, such as electrographic seizure cessation, avoidance of intubation, intensive care unit length of stay, and long-term neurological outcomes; and subgroup analyses based on etiology, age, and comorbidities to tailor therapeutic recommendations. Such trials would clarify Ketamine’s role in the SE algorithm and help define the patient populations most likely to benefit, such as those with BDZ-resistant SE and frail patients.

## Data Availability

Not applicable.

## Abbreviations

The following abbreviations are used in this manuscript:

Early SE	A status epilepticus stage requiring first-line therapy for ongoing seizure activity, typically with a benzodiazepine
Established SE	A stage with seizure activity persisting despite first line of treatment
SE	status epilepticus
RSE	Refractory status epilepticus
SRSE	Super-refractory status epilepticus
Anesthetics	Agents commonly used in continuous infusion after failure of at least one anti-seizure medication
ASM	Anti-seizure medication
BDZ	Benzodiazepine
NMDA	N-methyl-D-aspartate (NMDA) receptor of glutamate
AMPA	Alpha-amino-3-hydroxy-5-methyl-4-isooxazole-propionic acid glutamate receptors
CNS	Central nervous system

## References

[1] Schubert-Bast S., Zöllner J.P., Ansorge S., Hapfelmeier J., Bonthapally V., Eldar-Lissai A., Rosenow F., Strzelczyk A. (2019). Burden and epidemiology of status epilepticus in infants, children, and adolescents: A population-based study on German health insurance data. Epilepsia.

[2] Lu M., Faure M., Bergamasco A., Spalding W., Benitez A., Moride Y., Fournier M. (2020). Epidemiology of status epilepticus in the United States: A systematic review. Epilepsy Behav..

[3] Leitinger M., Trinka E., Zimmermann G., Granbichler C.A., Kobulashvili T., Siebert U. (2020). Epidemiology of status epilepticus in adults: Apples, pears, and oranges—A critical review. Epilepsy Behav..

[4] Bleck T.P. (2024). The future treatment of status epilepticus. Epilepsy Behav..

[5] Vossler D.G. (2025). First Seizures, Acute Repetitive Seizures, and Status Epilepticus. Continuum.

[6] Trinka E., Leitinger M. (2022). Management of Status Epilepticus, Refractory Status Epilepticus, and Super-refractory Status Epilepticus. Continuum.

[7] Glauser T., Shinnar S., Gloss D., Alldredge B., Arya R., Bainbridge J., Bare M., Bleck T., Dodson W.E., Garrity L. (2016). Evidence-Based Guideline: Treatment of Convulsive Status Epilepticus in Children and Adults: Report of the Guideline Committee of the American Epilepsy Society. Epilepsy Curr..

[8] Vossler D.G., Bainbridge J.L., Boggs J.G., Novotny E.J., Loddenkemper T., Faught E., Amengual-Gual M., Fischer S.N., Gloss D.S., Olson D.M. (2020). Treatment of Refractory Convulsive Status Epilepticus: A Comprehensive Review by the American Epilepsy Society Treatments Committee. Epilepsy Curr..

[9] Cornwall C.D., Krøigård T., Kristensen J.S.S., Callesen H.E., Beier C.P. (2023). Outcomes and Treatment Approaches for Super-Refractory Status Epilepticus: A Systematic Review and Meta-Analysis. JAMA Neurol..

[10] Horvat D.E., Keenan J.S., Javadian S., Liu Y.T., Voleti S., Staso K., Conley C., Schlatterer S.D., Sansevere A.J., Harrar D.B. (2025). Ketamine Versus Midazolam as the First-Line Continuous Infusion for Status Epilepticus in Children with Cardiac Disease. Neurocrit. Care.

[11] Caranzano L., Novy J., Rossetti A.O. (2022). Ketamine in adult super-refractory status epilepticus: Efficacy analysis on a prospective registry. Acta Neurol. Scand..

[12] Alkhachroum A., Der-Nigoghossian C.A., Mathews E., Massad N., Letchinger R., Doyle K., Chiu W.T., Kromm J., Rubinos C., Velazquez A. (2020). Ketamine to treat super-refractory status epilepticus. Neurology.

[13] Richards N.D., Howell S.J., Bellamy M.C., Beck J. (2025). The diverse effects of ketamine, jack-of-all-trades: A narrative review. Br. J. Anaesth..

[14] Buratti S., Giacheri E., Palmieri A., Tibaldi J., Brisca G., Riva A., Striano P., Mancardi M.M., Nobili L., Moscatelli A. (2023). Ketamine as advanced second-line treatment in benzodiazepine-refractory convulsive status epilepticus in children. Epilepsia.

[15] Chiriboga N., Spentzas T., Abu-Sawwa R. (2024). A systematic review and meta-analysis of ketamine in pediatric status epilepticus. Epilepsia.

[16] Othman A.A., Sadek A.A., Ahmed E.A., Abdelkreem E. (2025). Combined Ketamine and Midazolam Versus Midazolam Alone for Initial Treatment of Pediatric Generalized Convulsive Status Epilepticus (Ket-Mid Study): A Randomized Controlled Trial. Pediatr. Neurol..

[17] Burman R.J., Rosch R.E., Wilmshurst J.M., Sen A., Ramantani G., Akerman C.J., Raimondo J.V. (2022). Why won’t it stop? The dynamics of benzodiazepine resistance in status epilepticus. Nat. Rev. Neurol..

[18] Joshi S., Rajasekaran K., Hawk K.M., Chester S.J., Goodkin H.P. (2018). Status epilepticus: Role for etiology in determining response to benzodiazepines. Ann. Neurol..

[19] Kapur J., Elm J., Chamberlain J.M., Barsan W., Cloyd J., Lowenstein D., Shinnar S., Conwit R., Meinzer C., Cock H. (2019). Randomized Trial of Three Anticonvulsant Medications for Status Epilepticus. N. Engl. J. Med..

[20] Llauradó A., Quintana M., Ballvé A., Campos D., Fonseca E., Abraira L., Toledo M., Santamarina E. (2021). Factors associated with resistance to benzodiazepines in status epilepticus. J. Neurol. Sci..

[21] Rollo E., Romozzi M., Dono F., Bernardo D., Consoli S., Anzellotti F., Ricciardi L., Paci L., Sensi S.L., Della Marca G. (2023). Treatment of benzodiazepine-refractory status epilepticus: A retrospective, cohort study. Epilepsy Behav..

[22] Magro G. (2025). Early Polytherapy for Probably Benzodiazepine Refractory Naïve Status Epilepticus (Stage 1 Plus). Neurol. Int..

[23] Magro G., Laterza V. (2024). Status epilepticus: Is there a Stage 1 plus?. Epilepsia.

[24] Naylor D.E., Liu H., Niquet J., Wasterlain C.G. (2013). Rapid surface accumulation of NMDA receptors increases glutamatergic excitation during status epilepticus. Neurobiol. Dis..

[25] Niquet J., Nguyen D., de Araujo Furtado M., Lumley L. (2023). Treatment of cholinergic-induced status epilepticus with polytherapy targeting GABA and glutamate receptors. Epilepsia Open.

[26] Naylor D.E. (2023). In the fast lane: Receptor trafficking during status epilepticus. Epilepsia Open.

[27] Nusser Z., Sieghart W., Somogyi P. (1998). Segregation of different GABAA receptors to synaptic and extrasynaptic membranes of cerebellar granule cells. J. Neurosci..

[28] Venkatachalan S.P., Czajkowski C. (2012). Structural link between γ-aminobutyric acid type A (GABAA) receptor agonist binding site and inner β-sheet governs channel activation and allosteric drug modulation. J. Biol. Chem..

[29] Muir J., Arancibia-Carcamo I.L., MacAskill A.F., Smith K.R., Griffin L.D., Kittler J.T. (2010). NMDA receptors regulate GABAA receptor lateral mobility and clustering at inhibitory synapses through serine 327 on the γ2 subunit. Proc. Natl. Acad. Sci. USA.

[30] Li X., Zhou J., Chen Z., Chen S., Zhu F., Zhou L. (2008). Long-term expressional changes of Na^+^-K^+^-Cl^−^ co-transporter 1 (NKCC1) and K^+^-Cl^−^ co-transporter 2 (KCC2) in CA1 region of hippocampus following lithium-pilocarpine induced status epilepticus (PISE). Brain Res..

[31] Lee H.H., Jurd R., Moss S.J. (2010). Tyrosine phosphorylation regulates the membrane trafficking of the potassium chloride co-transporter KCC2. Mol. Cell. Neurosci..

[32] Lee H.H., Deeb T.Z., Walker J.A., Davies P.A., Moss S.J. (2011). NMDA receptor activity downregulates KCC2 resulting in depolarizing GABAA receptor-mediated currents. Nat. Neurosci..

[33] Kapur J., Coulter D.A. (1995). Experimental status epilepticus alters gamma-aminobutyric acid type A receptor function in CA1 pyramidal neurons. Ann. Neurol..

[34] Naylor D.E., Liu H., Wasterlain C.G. (2005). Trafficking of GABA(A) receptors, loss of inhibition, and a mechanism for pharmacoresistance in status epilepticus. J. Neurosci..

[35] Goodkin H.P., Yeh J.L., Kapur J. (2005). Status epilepticus increases the intracellular accumulation of GABAA receptors. J. Neurosci..

[36] Joshi S., Rajasekaran K., Hawk K.M., Brar J., Ross B.M., Tran C.A., Chester S.J., Goodkin H.P. (2015). Phosphatase inhibition prevents the activity-dependent trafficking of GABAA receptors during status epilepticus in the young animal. Epilepsia.

[37] Lattanzi S., Giovannini G., Brigo F., Orlandi N., Trinka E., Meletti S. (2023). Acute symptomatic status epilepticus: Splitting or lumping? A proposal of classification based on real-world data. Epilepsia.

[38] Urzì Brancati V., Pinto Vraca T., Minutoli L., Pallio G. (2023). Polymorphisms Affecting the Response to Novel Antiepileptic Drugs. Int. J. Mol. Sci..

[39] Niquet J., Baldwin R., Norman K., Suchomelova L., Lumley L., Wasterlain C.G. (2016). Midazolam-ketamine dual therapy stops cholinergic status epilepticus and reduces Morris water maze deficits. Epilepsia.

[40] Niquet J., Baldwin R., Norman K., Suchomelova L., Lumley L., Wasterlain C.G. (2017). Simultaneous triple therapy for the treatment of status epilepticus. Neurobiol. Dis..

[41] Dorandeu F., Dhote F., Barbier L., Baccus B., Testylier G. (2013). Treatment of status epilepticus with ketamine, are we there yet?. CNS Neurosci. Ther..

[42] Yılmaz G.B., Saraçoğlu K.T., Aykın U., Akça M., Demirtaş C., Saraçoğlu A., Yıldırım M. (2024). Efficacy of Low-Dose Ketamine and Propofol in the Treatment of Experimental Refractory Status Epilepticus on Male Rats. J. Neurosci. Res..

[43] Zhou R., Wang Y., Cao X., Li Z., Yu J. (2021). Diazepam Monotherapy or Diazepam-Ketamine Dual Therapy at Different Time Points Terminates Seizures and Reduces Mortality in a Status Epilepticus Animal Model. Med. Sci. Monit..

[44] Olney J.W., Labruyere J., Wang G., Wozniak D.F., Price M.T., Sesma M.A. (1991). NMDA antagonist neurotoxicity: Mechanism and prevention. Science.

[45] Paule M.G., Li M., Allen R.R., Liu F., Zou X., Hotchkiss C., Hanig J.P., Patterson T.A., Slikker W., Wang C. (2011). Ketamine anesthesia during the first week of life can cause long-lasting cognitive deficits in rhesus monkeys. Neurotoxicol. Teratol..

[46] Ikonomidou C., Bosch F., Miksa M., Bittigau P., Vöckler J., Dikranian K., Tenkova T.I., Stefovska V., Turski L., Olney J.W. (1999). Blockade of NMDA receptors and apoptotic neurodegeneration in the developing brain. Science.

[47] Huang T.-H., Lai M.-C., Chen Y.-S., Huang C.-W. (2023). The Roles of Glutamate Receptors and Their Antagonists in Status Epilepticus, Refractory Status Epilepticus, and Super-Refractory Status Epilepticus. Biomedicines.

[48] The Eclampsia Trial Collaborative G. (1995). Which anticonvulsant for women with eclampsia? Evidence from the Collaborative Eclampsia Trial. Lancet.

[49] Zeiler F.A., Matuszczak M., Teitelbaum J., Gillman L.M., Kazina C.J. (2015). Magnesium sulfate for non-eclamptic status epilepticus. Seizure Eur. J. Epilepsy.

[50] Kornhuber J., Bormann J., Hübers M., Rusche K., Riederer P. (1991). Effects of the 1-amino-adamantanes at the MK-801-binding site of the NMDA-receptor-gated ion channel: A human postmortem brain study. Eur. J. Pharmacol. Mol. Pharmacol..

[51] Kalemenev S.V., Zubareva O.E., Sizov V.V., Lavrent’eva V.V., Lukomskaya N.Y., Kim K.K., Zaitsev A.V., Magazanik L.G. (2016). Memantine attenuates cognitive impairments after status epilepticus induced in a lithium–pilocarpine model. Dokl. Biol. Sci..

[52] Nguyen L., Thomas K.L., Lucke-Wold B.P., Cavendish J.Z., Crowe M.S., Matsumoto R.R. (2016). Dextromethorphan: An update on its utility for neurological and neuropsychiatric disorders. Pharmacol. Ther..

[53] Tetz L.M., Rezk P.E., Ratcliffe R.H., Gordon R.K., Steele K.E., Nambiar M.P. (2006). Development of a rat pilocarpine model of seizure/status epilepticus that mimics chemical warfare nerve agent exposure. Toxicol. Ind. Health.

[54] Mody I., Pearce R.A. (2004). Diversity of inhibitory neurotransmission through GABA-A receptors. Trends Neurosci..

[55] Lévesque M., Avoli M. (2013). The kainic acid model of temporal lobe epilepsy. Neurosci. Biobehav. Rev..

[56] Akeju O., Song A.H., Hamilos A.E., Pavone K.J., Flores F.J., Brown E.N., Purdon P.L. (2016). Electroencephalogram signatures of ketamine anesthesia-induced unconsciousness. Clin. Neurophysiol..

[57] Green S.M., Roback M.G., Kennedy R.M., Krauss B. (2011). Clinical practice guideline for emergency department ketamine dissociative sedation: 2011 update. Ann. Emerg. Med..

[58] Chang L.C., Raty S.R., Ortiz J., Bailard N.S., Mathew S.J. (2013). The emerging use of ketamine for anesthesia and sedation in traumatic brain injuries. CNS Neurosci. Ther..

[59] Zeiler F.A., Teitelbaum J., West M., Gillman L.M. (2014). The ketamine effect on intracranial pressure in nontraumatic neurological illness. J. Crit. Care.

[60] Gaspard N., Foreman B., Judd L.M., Brenton J.N., Nathan B.R., McCoy B.M., Al-Otaibi A., Kilbride R., Fernández I.S., Mendoza L. (2013). Intravenous ketamine for the treatment of refractory status epilepticus: A retrospective multicenter study. Epilepsia.

[61] Bredmose P.P., Grier G., Davies G.E., Lockey D.J. (2009). Pre-hospital use of ketamine in paediatric trauma. Acta Anaesthesiol. Scand..

[62] Ilvento L., Rosati A., Marini C., L’Erario M., Mirabile L., Guerrini R. (2015). Ketamine in refractory convulsive status epilepticus in children avoids endotracheal intubation. Epilepsy Behav..

[63] Wellington N.J., Boųcas A.P., Lagopoulos J., Quigley B.L., Kuballa A.V. (2025). Molecular pathways of ketamine: A systematic review of immediate and sustained effects on PTSD. Psychopharmacology.

[64] Yan M., Sun T., Liu J., Chang Q. (2024). The efficacy and safety of ketamine in the treatment of super-refractory status epilepticus: A systematic review. J. Neurol..

[65] Ubogu E.E., Sagar S.M., Lerner A.J., Maddux B.N., Suarez J.I., Werz M.A. (2003). Ketamine for refractory status epilepticus: A case of possible ketamine-induced neurotoxicity. Epilepsy Behav..

[66] Prüss H., Holtkamp M. (2008). Ketamine successfully terminates malignant status epilepticus. Epilepsy Res..

[67] Strous J.F.M., Weeland C.J., van der Draai F.A., Daams J.G., Denys D., Lok A., Schoevers R.A., Figee M. (2022). Brain Changes Associated With Long-Term Ketamine Abuse, A Systematic Review. Front. Neuroanat..

[68] Fujikawa D.G. (2019). Starting ketamine for neuroprotection earlier than its current use as an anesthetic/antiepileptic drug late in refractory status epilepticus. Epilepsia.

[69] Jacobwitz M., Mulvihill C., Kaufman M.C., Gonzalez A.K., Resendiz K., Francoeur C., Helbig I., Topjian A.A., Abend N.S. (2024). A Comparison of Ketamine and Midazolam as First-Line Anesthetic Infusions for Pediatric Status Epilepticus. Neurocrit. Care.

[70] Jacobwitz M., Mulvihill C., Kaufman M.C., Gonzalez A.K., Resendiz K., MacDonald J.M., Francoeur C., Helbig I., Topjian A.A., Abend N.S. (2022). Ketamine for Management of Neonatal and Pediatric Refractory Status Epilepticus. Neurology.

[71] Fletman E.W., Cleymaet S., Salvatore A., Devlin K., Pickard A., Shah S.O. (2024). Ketamine plus midazolam compared to midazolam infusion for the management of refractory status epilepticus. Clin. Neurol. Neurosurg..

[72] Harnicher B., Murray N.M., Dresbach J., Collingridge D.S., Reachi B., Bair J., Hoang Q., Fontaine G.V. (2024). Ketamine reduces seizure and interictal continuum activity in refractory status epilepticus: A multicenter in-person and teleneurocritical care study. Neurol. Sci..

[73] Kimmons L.A., Alzayadneh M., Metter E.J., Alsherbini K. (2024). Safety and Efficacy of Ketamine Without Intubation in the Management of Refractory Seizures: A Case Series. Neurocrit. Care.

[74] Srinivas M., Parker D., Millis S., Marawar R., Zutshi D., Basha M.M. (2023). Factors Associated with Refractory Status Epilepticus Termination Following Ketamine Initiation: A Multivariable Analysis Model. Neurocrit. Care.

[75] Höfler J., Trinka E. (2018). Intravenous ketamine in status epilepticus. Epilepsia.

[76] Dzhala V.I., Talos D.M., Sdrulla D.A., Brumback A.C., Mathews G.C., Benke T.A., Delpire E., Jensen F.E., Staley K.J. (2005). NKCC1 transporter facilitates seizures in the developing brain. Nat. Med..

